# *Mycobacterium tuberculosis* Complex Mycobacteria as Amoeba-Resistant Organisms

**DOI:** 10.1371/journal.pone.0020499

**Published:** 2011-06-03

**Authors:** Felix Mba Medie, Iskandar Ben Salah, Bernard Henrissat, Didier Raoult, Michel Drancourt

**Affiliations:** 1 Unité de Recherche sur les Maladies Infectieuses et Tropicales Emergentes, UMR 6236 CNRS - Université de la Méditerranée, IRD 189, IFR 48, Faculté de Médecine, Marseille, France; 2 Architecture et Fonction des Macromolécules Biologiques, AFMB, UMR 6098 CNRS - Université de la Méditerranée, Marseille, France; Institut Pasteur, France

## Abstract

**Background:**

Most environmental non-tuberculous mycobacteria have been demonstrated to invade amoebal trophozoites and cysts, but such relationships are largely unknown for members of the *Mycobacterium tuberculosis* complex. An environmental source has been proposed for the animal *Mycobacterium bovis* and the human *Mycobacterium canettii*.

**Methodology/Principal Findings:**

Using optic and electron microscopy and co-culture methods, we observed that 89±0.6% of *M. canettii*, 12.4±0.3% of *M. tuberculosis*, 11.7±2% of *M. bovis* and 11.2±0.5% of *Mycobacterium avium* control organisms were phagocytized by *Acanthamoeba polyphaga*, a ratio significantly higher for *M. canettii* (*P* = 0.03), correlating with the significantly larger size of *M. canetti* organisms (*P* = 0.035). The percentage of intraamoebal mycobacteria surviving into cytoplasmic vacuoles was 32±2% for *M. canettii*, 26±1% for *M. tuberculosis*, 28±2% for *M. bovis* and 36±2% for *M. avium* (*P* = 0.57). *M. tuberculosis*, *M. bovis* and *M. avium* mycobacteria were further entrapped within the double wall of <1% amoebal cysts, but no *M. canettii* organisms were observed in amoebal cysts. The number of intracystic mycobacteria was significantly (*P* = 10^−6^) higher for *M. avium* than for the *M. tuberculosis* complex, and sub-culturing intracystic mycobacteria yielded significantly more (*P* = 0.02) *M. avium* organisms (34×10^4^ CFU/mL) than *M. tuberculosis* (42×10^1^ CFU/mL) and *M. bovis* (35×10^1^ CFU/mL) in the presence of a washing fluid free of mycobacteria. Mycobacteria survived in the cysts for up to 18 days and cysts protected *M. tuberculosis* organisms against mycobactericidal 5 mg/mL streptomycin and 2.5% glutaraldehyde.

**Conclusions/Significance:**

These data indicate that *M. tuberculosis* complex organisms are amoeba-resistant organisms, as previously demonstrated for non-tuberculous, environmental mycobacteria. Intercystic survival of tuberculous mycobacteria, except for *M. canettii*, protect them against biocides and could play a role in their life cycle.

## Introduction

Non-tuberculous mycobacteria, responsible for community-acquired and focal health-care associated infections following surgery and intervention procedures, emerged over the last decade. In France, *Mycobacterium xenopi* has been responsible for a large outbreak of rachis infections following vertebral disk surgery, as well as for cases of arthritis following joint surgery [1]. Likewise, *Mycobacterium abscessus* and *Mycobacterium chelonae* have been responsible for several outbreaks of surgical site infection following abdominoplasty and liposuction [2]. Also, an outbreak of *Mycobacterium fortuitum* furunculosis has been reported following shaving of the legs with a razor and a footbath in a nail salon [3]. Recently, large outbreaks of post-surgical infections caused by *Mycobacterium bolettii* and *Mycobacterium massiliense* involved several thousand patients in several hospitals in Brazil [4], [5].

In the aforementioned situations, infection was traced to improperly used water contaminated by non-tuberculous mycobacteria, illustrating that water was an important yet underestimated source for non-tuberculous mycobacteria. Indeed, the vast majority of non-tuberculous mycobacteria have been shown to be environmental organisms residing in soil and water [6]. It has been observed that such non-tuberculous mycobacteria could be recovered from water samples also colonized by free-living amoeba, such as a hospital water supply colonized by *M. xenopi* and a drinking water plant colonized by several species of non-tuberculous mycobacteria [7]. Additionally, non-tuberculous mycobacteria have been observed to reside within the cytoplasm of amoebal trophozoites, resulting in their characterization as amoeba-resistant organisms [8]. In amoeba, non-tuberculous mycobacteria could be eventually entrapped in the amoebal cyst, being uniquely located in the exocyst layer of the cyst wall [9]. These observations supported a model of a free-living amoebal cyst acting as a “Trojan horse” that protects embedded non-tuberculous, environmental mycobacteria against unfavorable conditions.

In contrast, the *Mycobacterium tuberculosis* complex (MTC) is uniquely comprised of seven closely related, host-associated organisms responsible for tuberculosis in mammals and humans with direct host-to-host transmission [10], [11]. MTC organisms have no known natural environmental stage, despite the fact that experimental data indicate that *M. tuberculosis* [12] and *Mycobacterium bovis* [13] could survive for hours to days in the amoebal trophozoites. These pioneering works did not firmly explore whether these MTC organisms could be further entrapped in the amoebal cyst, somewhat limiting interpretation of the data.

To gain further insight into the relationships between MTC organisms and free-living amoeba, we have quantified the relationships between representative organisms of the *M. canettii*, *M. tuberculosis* and *M. bovis* tuberculous species with the trophozoites and cysts of the free-living amoeba *Acanthamoeba polyphaga*.

## Materials and Methods

### *Mycobacterium* strains

*M. tuberculosis* H37Rv CIP103471, *M. canettii* CIP140010059^T^, *M. bovis* CIP671203 and a clinical isolate of *M. avium* subsp. *hominissuis* (herein referred to as *M. avium*) were subcultured on Middlebrook 7H10 agar (Becton Dickinson, Le Pont de Claix, France) for 21 days at 37°C in a 5% CO_2_ atmosphere. The *in vitro* susceptibility of these four organisms to 10 µg/mL streptomycin was determined by incubation on Middlebrook 7H9 and growth monitoring using the MGIT 960 apparatus (Becton Dickinson, Le-Pont-de-Claix, France). The susceptibility of *M. avium* and *M. tuberculosis* to glutaraldehyde was further tested by incubating *M. avium* and *M. tuberculosis* mycobacteria with 2.5% glutaraldehyde for 1 hour at room temperature, after which the cells were washed twice with phosphate buffered saline (PBS) and subcultured on Middlebrook 7H10 agar (Becton Dickinson) for 5 weeks at 37°C in a 5% CO_2_ atmosphere. Prior to infection, cells were harvested and dispersed by expelling the suspension 10 times through a sterile 25 gauge needle attached to a 1-mL syringe. The inoculum was adjusted to 10^6^ mycobacteria/ml in PBS. *A. polyphaga* Link-AP1 strain [14], amoeba were suspended twice in Page's modified Neff's Amoeba Saline (PAS) to obtain 5×10^5^ cells/mL, and 1 mL of such suspension was distributed into each well of a 12-well microplate (Corning, New York, USA) and inoculated with 100 µL of a 10^6^ mycobacteria/mL suspension (Multiplicity of infection (MOI) = 1/1) of either *M. tuberculosis* H37Rv, *M. canettii*, *M. bovis* or *M. avium*. The microplate was centrifuged at 1,000× *g* for 30 min and then incubated at 32°C under a humidified 5% CO_2_ atmosphere. The microplate was examined daily for 3 days for cytopathic effect. The presence of intra-amoebal organisms was determined by shaking, 10-min centrifugation at 200× g and observation using a light microscope after Ziehl-Neelsen staining. All experiments were done in triplicate.

### Encystment and excystment of infected amoeba

In 25-cm^2^ culture flasks (Corning, New York, USA), 10 mL of amoebal suspension infected for 24 hours was incubated with 10 µg/mL of streptomycin for 2 hours at room temperature to kill any remaining extra-amoebal and adherent mycobacteria. After treatment and washing, the trophozoites were rinsed with encystment buffer (0.1 M KCl, 0.02 M Tris, 8 mM MgSO_4_, 0.4 mM CaCl_2_ and 1 mM NaHCO_3_) and centrifuged at 1,000× g for 10 min, and the pellet was resuspended in 10 mL of fresh encystment buffer and incubated for three days at 32°C. Encystment of the amoeba was examined by light microscopy. After 3 days, amoebal cysts were pelleted by centrifugation at 1,000× g for 10 min. Some of the cysts were treated with 5 mg/mL of streptomycin for 48 hrs, and the remaining cysts were treated with 2.5% glutaraldehyde for 1 hour [15]. Treated cysts were washed three times with PAS. Some cysts were processed for electron microscopy (see below); the remainder was incubated for 7 days in PYG medium at 33°C. Intra-amoebal mycobacteria were released by lysing the monolayer with 1 mL of 0. 5% sodium dodecyl sulphate, followed by two successive passages through a 27-gauge needle [16]. The presence of viable mycobacteria was documented by detecting colonies on Middlebrook 7H10 agar inoculated with 100 µL of the cell lysate and incubated at 37°C for 21 days for MTC organisms and 15 days for *M. avium*.

### Ultrastructural studies

The size of the *Mycobacterium* organisms was determined by transmission electron microscopy observation of 21 organisms for each species to determine the median and standard deviation of cell length. Amoebal monolayers previously infected by MTC organisms were fixed in 2% glutaraldehyde and 0.1 M cacodylate buffer overnight, then in 2% glutaraldehyde and 0.33% acroleine in 0.07 M cacodylate buffer for 1 hour. After washing in 0.2 M cacodylate buffer, the preparation was fixed in 1% osmium tetraoxide with 0.1 M potassium ferrycyanure for 1 hour and dehydrated in an ascending series of ethanol concentrations, up to 100% ethanol. The samples were then successively incubated (for 45 min) in a 3∶1, 2∶2, 1∶3 (vol/vol) ethanol-Epon suspension, then in 100% Epon overnight with continuous shaking before being embedded in an Epon 812 resin (Fluka, St Quentin Fallavier, France) incubated for 3 days at 60°C. Ultrathin sections (70 nm) were cut from the blocks using an ultracut microtome (Reichert-Leica, Marseille, France) before being deposited on Formvar-coated copper grids (Sigma-Aldrich, Taufkirchen, Germany). Ultrathin sections were stained for 10 min. with 5% uranyl acetate and lead citrate before being examined using a transmission electron microscope (Morgani 268D; Philips, Eindhoven, the Netherlands).

### Measure of intraamoebal uptake and survival of mycobacteria

To measure the uptake of mycobacteria by *A. polyphaga* trophozoites, mycobacteria and amoeba were co-incubated at 37°C for 24 h prior to three washes with PAS. Washed trophozoites were then incubated in fresh medium containing 10 µg/mL of streptomycin for 2 hours to kill extracellular mycobacteria. The trophozoites were then washed three times with PAS and incubated at 37°C in fresh PAS. Intra-amoebal mycobacteria were released by lysing the monolayer with 1 ml of 0.5% sodium dodecyl sulphate, followed by two successive passages through a 27-gauge needle, and the lysates were plated as serial dilutions in complete Middlebrook 7H9 medium on Middlebrook 7H10 agar. Plates were incubated at 37°C for 21 days, and colony-forming units (CFUs) were counted. The intra-amoebal survival curves of *M. tuberculosis*, *M. canettii*, and *M. bovis* were determined after plating intra-amoebal mycobacteria after 0, 12, 24, 48, and 72 hours of infection.

### Statistical analyses

Data are expressed as means ± standard deviation; unpaired Student's t-test and one-way ANOVA test were used, and a *P* value<0.05 was considered significant.

## Results

### Uptake and survival of mycobacteria in trophozoites

**Figure 1:**
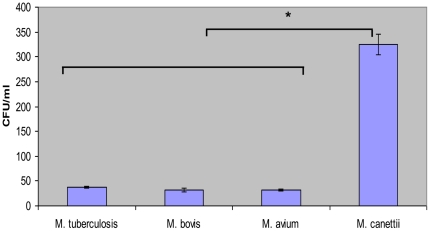
Uptake of mycobacteria by *A. polyphaga* trophozoites. Cells were exposed to mycobacteria for 24 h. “*” denotes a statistically significant difference.

**Figure 2:**
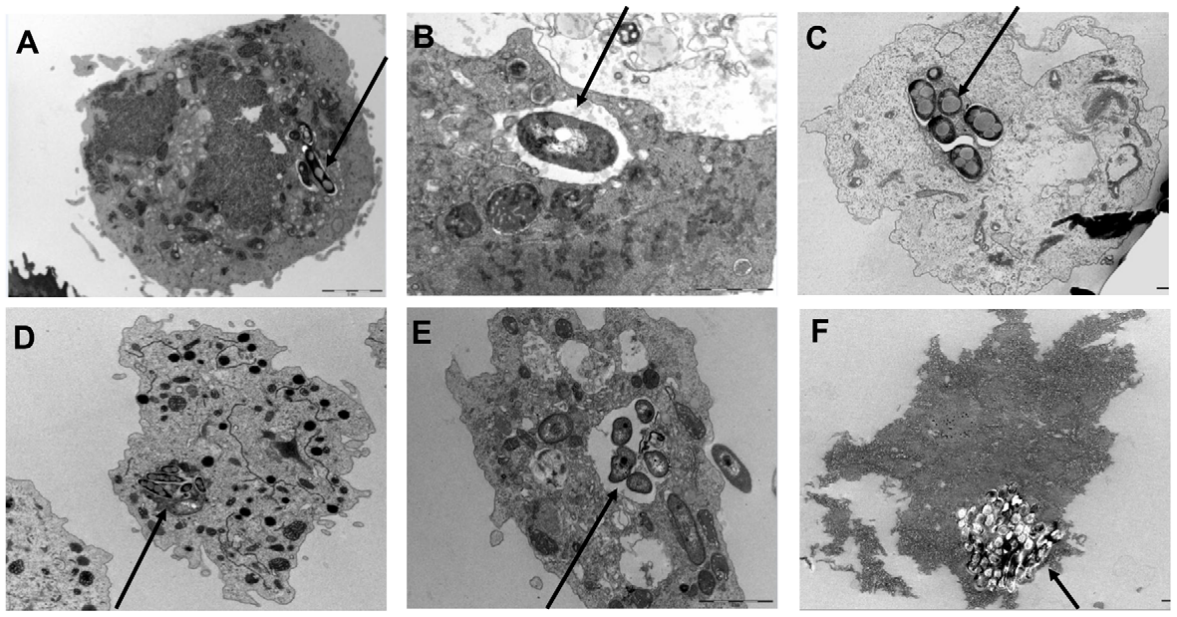
*M. tuberculosis* complex mycobacteria are internalized into amoeba. Transmission electron-microscopy observation of *M. tuberculosis* H37Rv (A and B), *M. bovis* (C), *M. avium* (D) and *M. canettii* (E and F) in *A. polyphaga* trophozoites.

**Figure 3:**
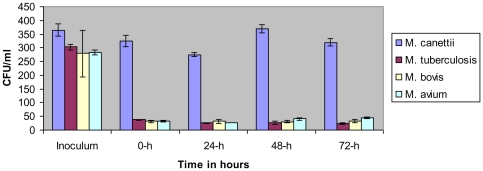
Survival of mycobacteria within *A. polyphaga* trophozoites at 33°C. Time zero represents 24 h after infection, immediately after treatment with streptomycin. Each bar represents the mean of duplicate wells, and error bars show the standard error of the mean. “*” denotes a statistically significant difference.

We observed that the length of mycobacterial cells used in this study was 2.56±0.5 µm for *M. canettii* organisms, 1.7±0.3 µm for *M. tuberculosis* organisms, 1.33±0.3 µm for *M. bovis* organisms, and 1.37±0.3 µm for *M. avium* organisms (Fig. S1); the length of *M. canettii* organisms was significantly larger than that of the three other mycobacteria (*P* = 0.035, one-way ANOVA). The four organisms were killed by 10 µg/mL streptomycin and *M. avium* and *M. tuberculosis* were killed also by 2.5% glutaraldehyde. Acid-fast stained *A. polyphaga* trophozoites exhibited intra-amoebal mycobacteria, regardless of the *Mycobacterium* species being studied. The proportion of inoculated mycobacteria phagocytozed by trophozoites was significantly higher (*P* = 0.03) for *M. canettii* organisms (89±0.6%) than for *M. tuberculosis* (12.4±0.3%), *M. bovis* (11.7±2%) and the *M. avium* control (11.2±0.5%) (Fig. 1). Phagocytosis yielded an insignificant difference in the percentage of infected amoeba, varying between 26±1% for *M. tuberculosis*, 28±2% for *M. bovis*, 32±2% for *M. canettii* and 36±2% for *M. avium*; the number of mycobacteria per trophozoite varied from 1 to 4 for *M. tuberculosis*, 1 to 6 for *M. bovis* and *M. avium* and from 1 to 10 for *M. canettii* (Fig. 2). Electron microscopy revealed that mycobacteria were residing inside one or several vacuoles without notable modification of the surrounding cytoplasm; vacuoles containing only one organism exhibited a close apposition of the vacuole membrane all over the mycobacterial cell surface (Fig. 2B). In some vacuoles, electron microscopy revealed morphological features that were compatible with mycobacterial division. Growth curves were not significantly different between the four species being studied, with the only observations being a small decrease in the number of CFUs at 24 h and minute variations in the number of CFUs from 24 h to 72 h (Fig. 3).

### Survival of mycobacteria in cysts

**Figure 4:**
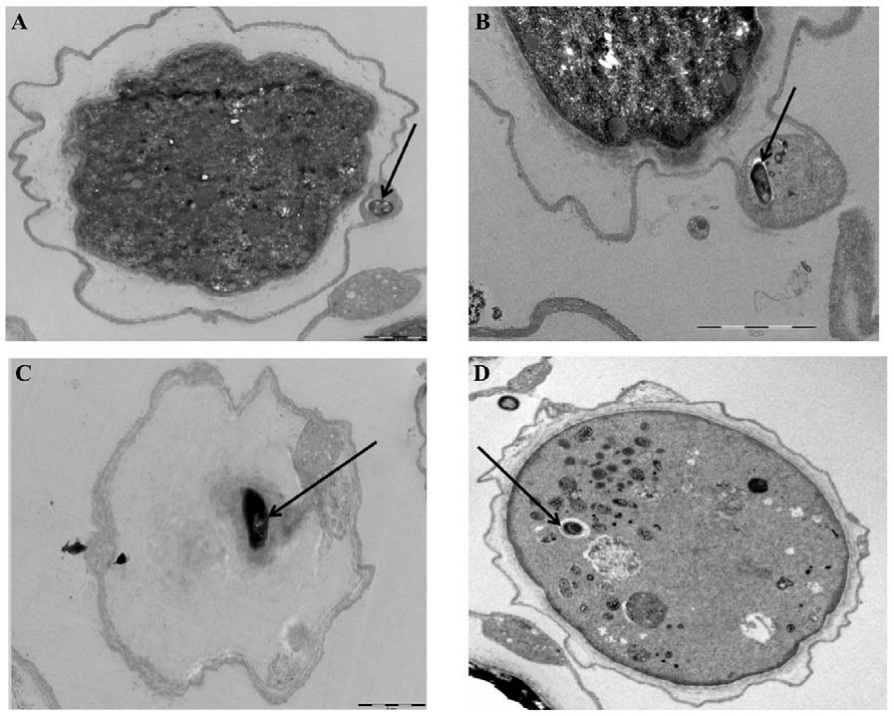
*M. tuberculosis* and *M. bovis*, but not *M. canettii* mycobacteria are included into amoebal cysts. Transmission electron-microscopy observation of *A. polyphaga* cysts containing *M. tuberculosis* H37Rv (A and B) within a double cell wall, *M. bovis* (C) and *M. avium* subsp. *hominissuis* (D) in the cytoplasm.

**Figure 5:**
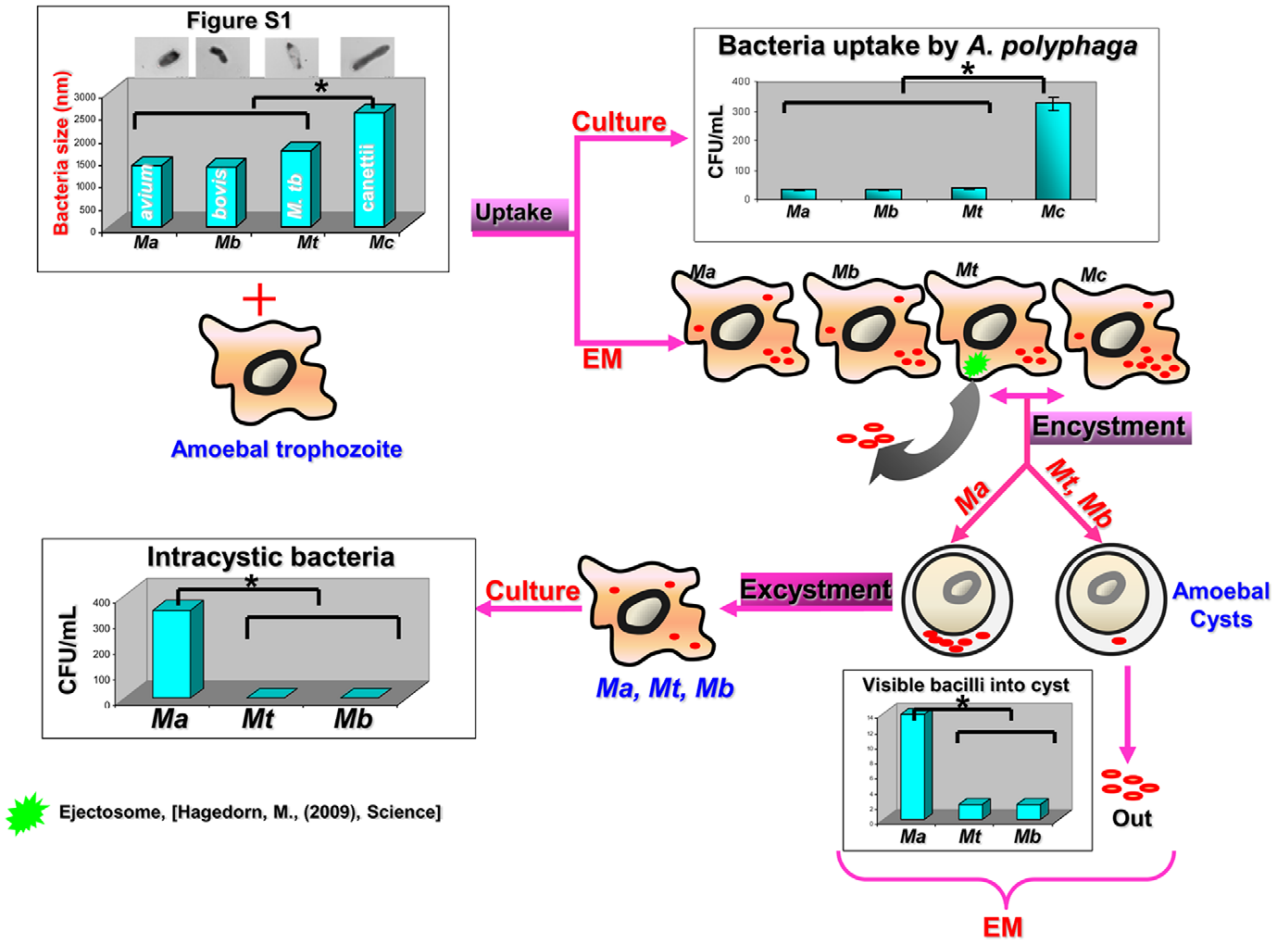
Difference in the mechanism of interaction between MTC and *M. avium*. Comparison of the size of mycobacteria by electron microscopy. *M. bovis* (A), *M. avium* (B), *M. tuberculosis* (C) and *M. canettii* (D). “*” denotes significant difference when comparing the size of *M. canettii* and that of the other species. Ma- *Mycobacterium avium*. Mb- *Mycobacterium bovis*. Mt- *Mycobacterium tuberculosis*. Mc- *Mycobacterium canettii*.

Light microscopy failed to reveal visible mycobacteria in *A. polyphaga* cysts three days after the induction of encystment, whereas electron microscopy observation of 300 such cysts showed *M. tuberculosis*, *M. bovis*, and *M. avium* organisms visible inside the *A. polyphaga* cysts (Fig. 4). As for these three species, we found <1% of cysts harboring mycobacteria visible in the space between the inner and outer wall of the cyst (Fig. 4A). *M. bovis* organisms were also observed on the inner side of the outer wall and in the cytoplasm of the cyst (Fig. 4C), as was the case for a few *M. avium* mycobacteria (Fig. 4D). The average number of mycobacteria per cyst was significantly higher (*P* = 10^−6^) for *M. avium* (5 bacilli/cyst) than for *M. tuberculosis* and *M. bovis* (1 bacillus/cyst). Further electron microscopy observation of up to 500 cysts failed to reveal any *M. canettii* organisms inside the *A. polyphaga* cysts. We further observed that a 48-hour exposure of the mycobacteria-infected cysts to 10 µg/ml streptomycin, used to kill extra-cystic mycobacteria, did not affect their excystment capacity, as new trophozoites emerged after a 7-day incubation in PYG medium at 33°C as determined by light microscopy. These cysts yielded significantly more (P = 0.02) *M. avium* organisms (34×10^4^ CFU/mL) than *M. tuberculosis* (42×10^1^ CFU/mL) and *M. bovis* (35×10^1^ CFU/mL) in the presence of a washing fluid free of mycobacteria (Fig. 5). After the cysts were exposed to 2.5% glutaraldehyde, sub-culturing intracystic mycobacteria released 18×10^3^ CFUs for *M. avium* and 220 CFUs for *M. tuberculosis*. A non-quantitative subculture further demonstrated that *M. avium*, *M. tuberculosis* and *M. bovis* organisms survived in the cysts of *A. polyphaga* for up to 18 days.

## Discussion

In this study, *M. avium*, used as a control organism to certify the validity of the experimental protocol, yielded results identical to those previously published showing that *M. avium* survived in *A. polyphaga* trophozoites and cysts, therefore validating our data obtained using MTC organisms [9]. We further observed a few *M. avium* organisms in the cytoplasm of *A. polyphaga* cysts, thus adding to published observations that this organism can exist between the inner and outer cyst walls [9], [17]. We ensured that the positive observations we made did not merely result from contamination, as we thoroughly washed inoculated amoeba and treated cysts with either 5 mg/mL streptomycin or 2.5% glutaraldehyde, a concentration that we controlled to be mycobactericidal for the MTC reference strains that were examined. The negative controls examined in successive phases of the study remained negative, further indicating the authenticity of our data.

Our observation that *M. tuberculosis* and *M. bovis* organisms were engulfed by *A. polyphaga* trophozoites agreed with previous observations made when co-culturing *M. tuberculosis* organisms with the free-living amoeba *Dictyostelium discodium* [12] and when co-culturing *M. bovis* organisms with *Acanthamoeba castellanii* [13]. Our observation that *M. canettii* organisms could also be ingested by amoebal trophozoites has not been previously studied; additionally, the ratio of phagocytosed *M. canettii* organisms was significantly higher than the phagocytosed ratio of other mycobacteria. Interestingly, *M. canettii* organisms were significantly larger than the two other MTC mycobacteria being studied, a previously unreported observation. The size of particles has been shown to determine the efficiency of phagocytosis by free-living amoeba, with a 0.5 µm minimum size for inert latex particles to be ingested by *Acantamoeba* amoeba [18]. *M. canettii* organisms exhibit other unique phenotypic traits, including the smoothest colonies, which could also influence amoebal phagocytosis [19], [20]. We propose that the size of mycobacterial cells is one of the determinants of their efficient ingestion by free-living amoeba.

All MTC organisms further survived in vacuoles, as previously demonstrated for *M. bovis* organisms in *A. castellanii* hosts for 14 days [13]; we presented data indicating that survival in the amoebal trophozoite is a common property for MTC members, which should be regarded as amoeba-resistant organisms [7]. Following phagocytosis, a significantly higher number of *M. canettii* organisms survived in the amoeba trophozoite. Such intra-amoebal survival is consistent with the theory that amoeba-resistant organisms evolved mechanisms allowing them to also penetrate and survive in macrophages, classifying them as pathogens [8]. Whereas *M. tuberculosis* and *M. bovis* organisms have been previously demonstrated to survive in the vacuoles of macrophages, a pivotal phenotype in the pathology of tuberculosis [21], [22], such survival has not been yet reported for *M. canettii*. Data presented in this study suggest that *M. canettii* would be also an organism surviving in macrophages in line with the observation that this organism is a very rare agent of human tuberculosis after its first isolation from a 20-year-old French patient [23]. Recognizing MTC organisms as amoeba-resistant organisms agrees with their closest genomic proximity with *Mycobacterium marinum*, a waterborne species also capable of survival within *D. discodium* and *A. polyphaga* [16], [24], [25]. The current evolutionary model of mycobacteria proposes that the most recent common ancestor (MRCA) for *M. tuberculosis* and *M. marinum* was a common generalist environmental *Mycobacterium* species [26]. Recent genome analysis of the environmental *Mycobacterium indicus pranii* enforced a scenario in which the MRCA was an aquatic species that gave rise to waterborne *M. marinum* and *Mycobacterium ulcerans* on one branch, the *Mycobacterium avium* complex on a second branch and the MTC on a third branch [27]. *Mycobacterium pinnepidii*, an MTC organism responsible for tuberculosis in seals, indeed retained an aquatic habitat [28], [29]. In this scenario, intra-amoebal life of MRCA may have selected traits in MTC organisms that endowed them with resistance to macrophages. Such intra-amoebal survival could be exploited for laboratory diagnosis of tuberculosis from contaminated specimens; amoeba are able to allow for the survival of host-adapted organisms while destroying contaminant organisms, as previously reported for the initial recovery of the non-tuberculous *Mycobacterium massiliense* from human sputum [30]. Indeed, we have been able to isolate *M. tuberculosis* organisms from various contaminated clinical specimens using *A. polyphaga* as a cleaning scavenger (D. Raoult, unpublished data).

We further observed that a few *M. tuberculosis* and *M. bovis* organisms were entrapped in the amoebal cyst, more precisely located between the two external layers of the amoebal cyst, a property previously reported as a hallmark of mycobacteria among amoeba-resisting organisms [9]. However, extensive electron microscope observation failed to reveal any *M. cannetii* organisms in cysts and only an average number of 1 *M. tuberculosis* and *M. bovis* bacillus per infected cyst. This was in significant contrast with *M. avium*, which yielded an average of 5 visible bacilli per infected cyst, resulting in the fact that *M. avium* yielded 2 logs more viable mycobacteria than MTC organisms after the excystment of infected cysts. These data extend a previous preliminary study in which the ratio of encystment (approximately 50%) was not driven by the experimental conditions and in which *M. bovis* organisms were not observed in cysts [13]. Therefore, later experiments did not provide clear-cut evidence for the survival of *M. bovis* organisms in amoebal cysts. Combining morphological and cultural data indicates that, in contrast to *M. avium*, the majority of MTC organisms bypass the amoebal cyst after they are phagocytosed into the amoebal trophozoite. These data agree with the previous demonstration that *M. tuberculosis* organisms, but not *M. avium* organisms, were ejected from the amoeba *Dictyostelium* trophozoite by using an actin-based ejectosome [12]. MTC organisms may rely on their unique cellulase equipment, which are sugar cleaving enzymes capable of hydrolysing cellulose, a major component of the amoebal cyst cell wall [31], [32], to bypass amoebal cysts; the MTC organisms which still resided into the amoebal cyst, did resist glutaraldehyde, a biocide used to decontaminate medical devices, after entrapment in the amoebal cyst.

In conclusion, MTC should be regarded as amoeba-resistance organisms with the amoebal cyst protecting them against biocides except for *M. canettii*. These data may have implications for understanding the natural cycle of some MTC organisms.

## Supporting Information

Figure S1 **Comparison of the size of mycobacteria by electron microscopy.** *M. bovis* (A), *M. avium* (B), *M. tuberculosis* (C) and *M. canettii* (D). There was a significant difference when comparing the size of *M. canettii* and that of the other species, (P = 0. 035), as assessed by unpaired Student's t test and one way ANOVA.
(TIF)
