# Supplementary figures and images for: Mycobacterium tuberculosis Complex Mycobacteria as Amoeba-Resistant Organisms
Source: PLoS One. 2011 Jun 3;6(6):e20499. doi: 10.1371/journal.pone.0020499 (PMC3108610; doi:10.1371/journal.pone.0020499)

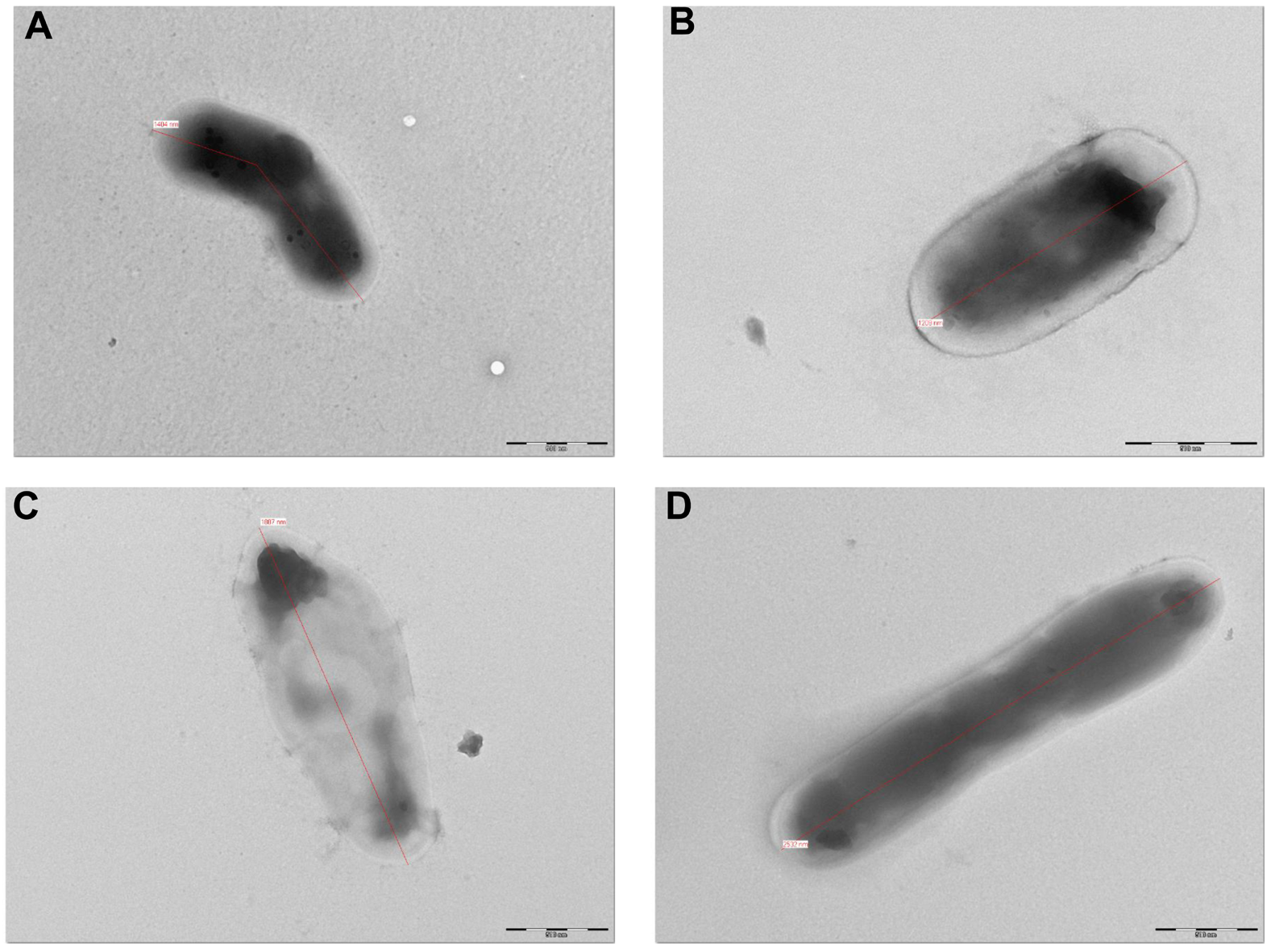

Supplement: Figure S1 — Comparison of the size of mycobacteria by electron microscopy. M. bovis (A), M. avium (B), M. tuberculosis (C) and M. canettii (D). There was a significant difference when comparing the size of M. canettii and that of the other species, (P = 0. 035), as assessed by unpaired Student's t test and one way ANOVA. (TIF) [file pone.0020499.s001.tif]
